# Psychometric Properties of Flourishing Scales From a Comprehensive Well-Being Assessment

**DOI:** 10.3389/fpsyg.2021.652209

**Published:** 2021-04-21

**Authors:** Dorota Weziak-Bialowolska, Piotr Bialowolski, Matthew T. Lee, Ying Chen, Tyler J. VanderWeele, Eileen McNeely

**Affiliations:** ^1^Sustainability and Health Initiative (SHINE), Department of Environmental Health, Harvard T. H. Chan School of Public Health, Boston, MA, United States; ^2^Human Flourishing Program, Institute for Quantitative Social Science, Harvard University, Cambridge, MA, United States; ^3^Department of Epidemiology, Harvard T. H. Chan School of Public Health, Boston, MA, United States

**Keywords:** multidimensional well-being, human flourishing, financial security, health, psychometric analysis, meaning and purpose, character strengths, social connectedness

## Abstract

In this article, we develop a measure of complete well-being. The framework is derived from the theoretical model of human flourishing understood as a state in which all aspects of a human life are favorable. The approach extends beyond psychological well-being and reflects the World Health Organization definition of health that not only considers the health of body and mind but also embraces the wholeness of the person. The Well-Being Assessment (WBA) is a comprehensive instrument designed to assess holistic well-being in six domains: emotional health, physical health, meaning and purpose, character strengths, social connectedness, and financial security. Although each of these domains is distinct, all of them are nearly universally desired, and all but financial security constitute ends in themselves. Data were collected from a representative sample of working adults. A sample of 276 employees participated in the pilot, 2,370 participated in the first wave and 1,209 in the second wave of the survey. The WBA showed a good fitting (40 items, six factors), satisfactory reliability, test–retest correlation, and convergent/discriminant validity in relation to stability over time and relevant health measures, as well as a good fit to the data that were invariant over time, gender, age, education, and marital status. The instrument can be of use for scientists, practitioners, clinicians, public health officials, and patients. Adoption of more holistic measures of well-being that go beyond psychological well-being may help to shift the focus from health deficiencies to health and well-being promotion.

## Introduction

Numerous definitions and survey measures of well-being and flourishing have been proposed (Hone et al., 2014; Su et al., 2014). Their core foundation is happiness, life satisfaction, and positive affect. A sense of meaning and/or purpose and other aspects of eudaimonic well-being [i.e., fulfillment of human potential and a meaningful life resulting from self-truth and self-responsibility (Norton, 1976; Ryan and Deci, 2001)] are also frequently included in composite well-being (flourishing) measures (Ryff and Keyes, 1995; Ryff and Singer, 2008). A comparison of four prominent measures of “flourishing” conducted by Hone et al. (2014); for a review of measures of “psychological well-being” and “thriving,” see Su et al. (2014), involving the conceptualizations and operationalizations of Keyes (2002, 2007), Diener et al. (2010), Huppert and So (2013), and Seligman (2011), revealed that all approaches included items referring to positive relationships, positive affect or engagement, and purpose or meaning (two instruments included both). Three out of four included self-acceptance and/or self-esteem, as well as competence or accomplishment. Two covered optimism and social contribution. Only one of the four measures included social integration, social growth, social acceptance, social coherence, environmental mastery, personal growth, autonomy, emotional stability, vitality, and resilience. Despite their important role for complete well-being, measures of well-being/flourishing usually neglect financial issues (Bialowolski et al., 2021). They also rarely focus on physical health. Character strengths are marginally present only in Seligman's (2011) accomplishment domain of the PERMA model of flourishing (P stands for positive emotion; E for engagement; R for relationships; M for meaning; and A for accomplishments). Even the 58-item, 18-domain “Comprehensive Inventory of Thriving” (Su et al., 2014) omits physical health and finances and only indirectly assesses character. With such a diversity, it is no wonder that findings generated by aggregated scales are inconsistent and difficult to reconcile, and a consensus in the field is needed (Hone et al., 2014).

Consequently, there seems to be a space for expanding the scope of well-being measurement. In particular, as argued by VanderWeele (2017), if the intention is to assess complete human well-being or human flourishing with a composite measure, features beyond psychological well-being should be considered. In this vein, a concept of complete well-being, which originates from the theoretical model of human flourishing understood as a state in which all aspects of a human life are good, has been proposed (VanderWeele, 2017; VanderWeele et al., 2019). This conceptual framework includes not only commonly agreed domains of flourishing (e.g., happiness, life satisfaction, affect, meaning and purpose, and social relationships) but also those that are often neglected in other measures. Regarding the latter, first, the framework assumes that domains such as physical health as well as character and virtue should become an inherent part of the composite measure. These domains, despite being analyzed as outcomes (e.g., physical health) or contributors (e.g., character strengths) to well-being, are seldom included as components of well-being measures. Second, it is also recognized that financial and material resources are necessary in order to achieve or sustain complete well-being over time, and thus an inclusion of a financial security domain in the assessment is proposed.

In order to overcome the current issues in measurement of well-being (Diener and Seligman, 2004; Lee et al., 2021b), the Well-Being Assessment (WBA) was proposed by the Human Flourishing Program and the Sustainability and Health Initiative for NetPositive Enterprise (SHINE) at Harvard University. The aim was to develop a measure of complete well-being that produces reliable and valid scores on each of its domains. The measure comprises six theoretically coherent and interrelated, yet distinct, domains of well-being: emotional health, physical health, social connectedness, meaning and purpose, character strengths, and financial security (see VanderWeele, 2017 for the rationale for these domains). Consequently, the concept of complete well-being, or human flourishing applied in this study, is in line with the World Health Organization definition of health^1^ that not only considers the health of body and mind but also embraces the wholeness of the person.

The WBA's logic follows the top-down (dispositional) hypotheses of well-being (Diener, 1984; Diener et al., 1999) that assume that because each person tends to evaluate her or his experiences in a positively or negatively oriented way (Judge and Hulin, 1993), well-being is reflected in its domains. Therefore, the WBA was conceptualized according to the logic of reflective measurement (Bagozzi, 2007; Howell et al., 2007) and developed focusing simultaneously on ensuring satisfactory psychometric properties of each of the WBA domains (as they may perhaps be used as independent measures in future research) as well as of the WBA itself (Furr, 2011).

## Conceptual Framework for Well-Being Assessment

The conceptualization of WBA in the present study originates from VanderWeele's (2017) definition of human flourishing and his Secure Flourishing Index (SFI). He defines flourishing in terms of complete human well-being and distinguishes its six domains: (1) life satisfaction and happiness, (2) meaning and purpose in life, (3) mental and physical health, (4) satisfactory relationships, (5) character and virtue, and (6) financial and material stability. By offering an expanded list of items covering in greater detail on the six domains of the SFI, the WBA provides more depth and nuance about crucial aspects of flourishing that are “ends” in themselves and that represent a nearly universal consensus of the meaning of complete well-being (Lee et al., 2021a). However, in this study, the composition of SFI was refined and the labeling of well-being domains was reworded as suggested by a group of well-being experts involved in this study. Thus, the instrument appeared to be of greater practical utility in a workplace setting focused on the well-being of employees. First, the number of items was expanded from 12 to 45 (initially; in the following steps, this number was reduced to 40) to gain an understanding of a broader range of aspects present in each of the domains of the SFI. Second, although the SFI combines mental and physical health in a single domain, recent empirical research suggested separating mental and physical health domains, whereas mental health and happiness/life satisfaction domains could be combined (Lee et al., 2021a). Consequently, the WBA was developed to comprise the following six domains.

### Emotional Health

Emotional health was conceptualized to focus on hedonic sentiments such as feeling happy and being satisfied with life. It also comprised such elements as having good mental health and being in control and able to deal with difficult emotions. Consequently, this domain was designed to cover judgments of overall life evaluations focusing on emotional and mental aspects of human functioning as well as emotional autonomy and resilience (Diener, 1984; Ryff and Keyes, 1995; Cohen and Pressman, 2006; Cohn et al., 2009; Diener et al., 2012).

### Physical Health

Physical health was defined by encompassing evaluations of physical functioning, healthy practices, and health maintenance. Its measurement focused on a comprehensive assessment of being sufficiently healthy to be able to carry out important tasks in life at present and in the future (Cho et al., 2011; Hernandez et al., 2018).

### Meaning and Purpose

This eudaimonic measure of well-being (National Research Council, 2013) was designed to reflect the value of one's life and to include elements of sense of meaning in life. Additionally, having direction to one's activities, pursuit of what is most important in life, and transcendence were included in the dimension. Consequently, this domain comprised an existential dimension referring to overall relatedness, coherence, and significance of personal experiences, as well as goal-oriented concepts related to pursuit and aspiration of certain ends (Reker et al., 1987; King et al., 2006; George and Park, 2013; Bronk, 2014).

### Character Strengths

This domain was conceptualized according to the philosophical and religious conviction, recently adopted by positive psychology, that in order to attain complete well-being, an excellent character and acting in accordance with the virtue, are essential (Peterson and Seligman, 2004; Seligman et al., 2005; Aristotle and Brown, 2009; Huber et al., 2019). Consequently, this domain was defined as an ability to focus, maintain consistent thoughts, and act in a way that contributes to the good of oneself and others, therefore comprising elements of virtuous decision making and altruism, perseverance and delayed gratification, and other character strengths.

### Social Connectedness

This domain focuses on the quantity and quality of desired and experienced social connections. Given the potential discrepancy between the number and quality of relationships that one maintains and desires, as argued in the cognitive discrepancy theory of loneliness (Peplau and Perlman, 1979; Perlman and Peplau, 1981), this domain also comprises elements of loneliness, which refer to a social deficiency (Campaign to End Loneliness, 2016). Finally, recognizing the importance of feeling respected by and connected to community, as well as having relationships that are close, meaningful, and supportive, this domain was designed to include, on the one hand, elements of social connection, support and intimacy, and, on the other hand, of communal social well-being (Haller and Hadler, 2006; Cruwys et al., 2013; Ambrey et al., 2017).

### Financial Security

This domain was designed to feature the sustainability and resilience of flourishing. It was intended to underscore that both sufficiently stable financial and material resources should be ensured to achieve, preserve, and then enhance complete well-being. Consequently, this domain was defined to comprise self-assessments of sufficient financial resources, and financial capability to be able to pursue one's life goals and not overly worry about making ends meet (Prawitz et al., 2006; Taylor et al., 2011; Białowolski et al., 2019).

While the first three domains and the last one have an individual focus, social well-being and some items in the character strengths domain transcend self-interest and connect to communal well-being (VanderWeele, 2019). The full list of questions is presented in Table 1. Based on the literature review, we also recognized that some of the suggested WBA domains are not well-represented in taxonomies of positive psychology, positive health, and ill-being. However, previous studies suggest that these domains should be considered relevant for well-being. Specifically, by directly asking respondents to evaluate importance of various well-being components for complete flourishing, Lee et al. (2021a) found that the average self-rated importance of six flourishing domains, that is, emotional health, physical health, meaning and purpose, character strengths, social connectedness, and financial security, was 8.97 (SD = 1.1) on a 0–10 scale. Physical health was rated as most important (9.33) and social connectedness as the least important (8.63) (Lee et al., 2021a), though most respondents believed in the importance of all domains.

**Table 1:** Six Well-Being Assessment components and 40 items.

**Domain**	**Code**	**Question and response scale**
Emotional health	EH1	Overall, how satisfied are you with life as a whole these days? (0 = Not satisfied at all, 10 = Completely satisfied)
Emotional health	EH2	On average, how happy have you felt during the last 7 days? (0 = Extremely unhappy, 10 = Extremely happy)
Emotional health	EH3	I expect more good things in my life than bad (0 = Strongly disagree, 10 = Strongly agree)
Emotional health	EH4	How would you rate your overall mental health? (0 = Poor, 10 = Excellent)
Emotional health	EH5	Are you depressed? (0 = Not at all depressed, 10 = Very depressed) (r)
Emotional health	EH6	Do you have anxiety that keeps you from doing the things in life that you need to do? (0 = Never, 10 = Always) (r)
Emotional health	EH7	In stressful situations, I manage my emotions so that I am still in control of myself (0 = Strongly disagree, 10 = Strongly agree)
Physical health	PH1	In general, how would you rate your physical health? (0 = Poor, 10 = Excellent)
Physical health	PH2	I have no major illnesses or injuries (0 = Strongly disagree, 10 = Strongly agree)
Physical health	PH3	I do not routinely get sick (0 = Strongly disagree, 10 = Strongly agree)
Physical health	PH4	My health does not prevent me from doing what I would like (0 = Strongly disagree, 10 = Strongly agree)
Physical health	PH5	My pain makes it hard for me to do my usual activities (0 = Strongly disagree, 10 = Strongly agree) (r)
Physical health	PH6	Based on my past health, I expect to be healthy long into the future (0 = Strongly disagree, 10 = Strongly agree)
Physical health	PH7	I regularly do things to maintain and improve my health, in diet, exercise, and health care (0 = Strongly disagree, 10 = Strongly agree)
Meaning and purpose	MP1	I know what gives meaning to my life (0 = Strongly disagree, 10 = Strongly agree)
Meaning and purpose	MP2	I have values and beliefs that help me understand who I am (0 = Strongly disagree, 10 = Strongly agree)
Meaning and purpose	MP3	My life has a clear sense of purpose (0 = Strongly disagree, 10 = Strongly agree)
Meaning and purpose	MP4	I understand my purpose in life (0 = Strongly disagree, 10 = Strongly agree)
Meaning and purpose	MP5	Overall, to what extent do you feel the things you do in your life are worthwhile? (0 = Not at all worthwhile, 10 = Completely worthwhile)
Meaning and purpose	MP6	I am pursuing what is most important to me in my life (0 = Strongly disagree, 10 = Strongly agree)
Character strengths	CS1	I always act to promote good in all circumstances, even in difficult and challenging situations (0 = Not true of me, 10 = Completely true of me)
Character strengths	CS2	I always know the right thing to do (0 = Not true of me, 10 = Completely true of me)
Character strengths	CS3	I always treat everyone with kindness, fairness, and respect (0 = Not true of me, 10 = Completely true of me)
Character strengths	CS4	I am always able to give up some happiness now for greater happiness later (0 = Not true of me, 10 = Completely true of me)
Character strengths	CS5	I am willing to face difficulties in order to do what is right (0 = Not true of me, 10 = Completely true of me)
Character strengths	CS6	I give up personal pleasures whenever it is possible to do some good instead (0 = Not true of me, 10 = Completely true of me)
Character strengths	CS7	I get to use my strengths to help others (0 = Not true of me, 10 = Completely true of me)
Social connectedness	SC1	My relationships are as satisfying as I would want them to be (0 = Strongly disagree, 10 = Strongly agree)
Social connectedness	SC2	There are people who really understand me (0 = Never, 10 = Always)
Social connectedness	SC3	How often do you feel lonely? (0 = Never, 10 = Almost always) (r)
Social connectedness	SC4	I am content with my friendships and relationships (0 = Strongly disagree, 10 = Strongly agree)
Social connectedness	SC5	I have enough people I feel comfortable asking for help at any time (0 = Strongly disagree, 10 = Strongly agree)
Social connectedness	SC6	I feel connected to the broader community around me (0 = Strongly disagree, 10 = Strongly agree)
Social connectedness	SC7	People in my broader community trust and respect one another (0 = Strongly disagree, 10 = Strongly agree)
Financial security	FS1	I am able to meet my normal monthly living expenses without any difficulty (0 = Completely disagree, 10 = Completely agree)
Financial security	FS2	How often do you worry about food, housing, or health expenses? (0 = Do not ever worry, 10 = Worry all of the time) (r)
Financial security	FS3	I have sufficient savings that I could cover 6 months of expenses (0 = Strongly disagree, 10 = Strongly agree)
Financial security	FS4	My financial circumstances give me freedom to pursue my goals (0 = Strongly disagree, 10 = Strongly agree)
Financial security	FS5	Given my age, I have done adequate financial planning for the future (0 = Strongly disagree, 10 = Strongly agree)
Financial security	FS6	The amount of debt I have often overwhelms me (0 = Strongly disagree, 10 = Strongly agree) (r)

*(r) reverse-coded item*.

## Materials and Methods

### Development and Refinement of the Item Pool for Well-Being Assessment

The WBA was designed to consist of six domains: emotional health, physical health, meaning and purpose, character strengths, social connectedness, and financial security. Preliminary development and refinement of the 45-item pool started with the definitions of the six domains (presented above). Subsequently, the research team comprising academics and well-being practitioners conducted an extensive review of well-being related literature in search for items already established in similar contexts. The aim was to include items that had been already frequently used in surveys, polls, and other studies. The approach drew upon prior empirical validation of specific questions and supplemented the initial pool of items with items that were either constructed or adapted from existing instruments. Thus, it was possible to better elucidate the conceptual space of each domain described above.

Through a multi-meeting process of item selection, refinement, and deletion, followed by pilot testing in focus groups, and then electronically administered pilot testing (in February 2018; T0) to a sample of 276 employees at a large national employer based in the United States, a set of 45 items was initially selected comprising questions from the six domains described above.

The subsequent, yet still preliminary, analyses based on a 45-item set (presented in the Supplementary Material 1, Tables A1–A3) aimed to reduce the item pool by retaining items that (1) loaded substantially on the factor they were designed to measure; (2) contributed to the reliability of the scale; and (3) were associated with the largest extent with their corresponding and theoretically relevant domain controlling for a rich set of demographic, socioeconomic, and lifestyle-related covariates. The goal was also to retain items that maintained the breadth of the original domain as well as items of the original SFI. Accordingly, a total of 40 items were selected across the six domains described above. Seven items were selected for each of the domains of emotional health, physical health, character strengths, and social connectedness. To measure meaning and purpose as well as financial security, sets of six items were used. The detailed structure of the WBA is presented in Table 1.

In the subsequent steps, two additional data collections were administered. Data were again collected from employees of the same employer in June 2018 (Time 1, henceforth also referred to as T1) and in July 2019 (Time 2; T2). All analyses presented henceforth are based on the set of 40 items administered at T1 and T2.

### Participants

Data were collected from a representative sample of working adults employed at a large U.S. company. A sample of 276 employees participated in the pilot, 2,370 participated in the first wave (T1) and 1,209 (out of 2,370 participating at T1) in the second wave (T2) of the survey. The survey was designed to comprehensively assess multidimensional human flourishing among workers. Data collection was preceded by a communication campaign 1 week prior to the survey administration to invite employees to participate in the survey. The survey was administered online to allow participants report on sensitive health and well-being topics in a secure and anonymous space of their choice. All current employees at least 18 years of age were eligible to participate in the survey. Participation was voluntary and confidential. Informed written consent was obtained from the participants. All protocols for recruitment and participation were reviewed and approved by the Harvard Longwood Campus Institutional Review Board.

The initial invitation and reminders to participate at T1 were sent to 15,000 employees through the work email system. A cash prize ranging from $100 to $1,000 was offered as an incentive to 52 randomly selected respondents out of those participating in each wave. The invitation and reminders to participate at T2 were sent to T1 participants only.

Along with well-being self-reports and well-being while-at-work assessments, all participants provided basic demographic information (i.e., age, gender, race, marital status, number of minor children at home, education level, ethnic background, voting behavior, and home ownership). Additionally, they reported on spiritual practices, religious service attendance, and volunteering, which previous research showed to be relevant for well-being (Fisher, 2000; Jenkinson et al., 2013; VanderWeele et al., 2016; Pawlikowski et al., 2019). There were only minor differences between the demographic profiles of the samples used in T1 and T2. Consistent with the largely female workforce at the organization, of the total of 2,370 T1 participants, 82% were female, 14% were at most 30 years old, 29% were from the 31–40 age group, 29% from the 41–50 age group, and 29% were older than 50 years. Of the total 1,209 T2 participants, 85% were female, 9% were at most 30 years old, 28% were from the 31–40 age group, 30% from the 41–50 age group, and 32% were older than 50 years.

In total, we considered 3,579 sets of responses provided by 2,370 distinct individuals. We divided them into two groups: Group 1 comprising 1,161 participants who completed the WBA survey at T1 only and Group 2 comprising 1,209 participants who completed surveys at T1 and T2. Different sets of analyses were conducted on different groups of respondents. Whenever responses from T1 and T2 were pooled, we used a complex design option to adjust standard errors for the fact that there were two sets of responses for the 1,209 participants (Muthén and Muthén, 2012).

### Measures: Key Correlates of Six Domains of Well-Being Assessment

#### Objective Measures of Health

Two mental health and two physical health outcomes derived from participants' medical insurance claims data at T2 were used as an external criterion for emotional health and physical health, respectively. These were actual diagnoses of depression, anxiety, obesity, and migraines and other headaches. It was presumed that they would be negatively associated with emotional health (depression and anxiety) and physical health (obesity and migraines).

Health insurance data record diagnostic information on medical conditions and treatments given, along with financial measures such as billed amounts, reimbursed amounts, and patient cost sharing. The health conditions in the claims data are determined based on the International Classification of Diseases (ICD-10) (WHO, 2004). Diagnostic information of the insurance claims data has been already shown to closely reflect actual medical records (Quam et al., 1993; Tyree et al., 2006).

#### Self-Reported Measures of Health

Four self-reported measures of health from the corporate health risk assessment conducted by the employer at T2 were used. Two indicators of emotional health comprised self-reports of (1) the negative affect (Have you felt down, depressed, or hopeless in the past 2 weeks? Response scale: yes/no) and (2) the stress level (How is your stress level? Response scale: no stress at all or low level, moderate level, and high level). They were expected to negatively correlate with emotional health and likely with physical health (also negatively). Additionally, a single general health question about self-assessments of health was used (In general, would you say that your health is? Response scale: Poor, fair, good, very good, and excellent). This measure was expected to positively correlate with physical and emotional health domains. Additionally, a question about the number of hours of sleep (How many hours of sleep do you usually get each day? Response scale: <7, 7–9, and more than 9 h) was used as an indicator of general health, since sleep difficulties can be linked to both physical and emotional problems (Kim et al., 2015; Simon and Walker, 2018).

Corporate health risk assessment is usually conducted on an annual basis. Despite varying in design, a corporate health assessment tool usually takes the form of a questionnaire and is used by employers to monitor organizational performance measured in terms of their populations' health as well as their corporate health policies and programs (Fabius et al., 2018). Prior studies have shown that investment in workforce health and well-being and consequently incorporating health and safety metrics (i.e., health risk assessment) when measuring corporate performance may help organizations establish a culture of health in the workplace and improve financial performance (Goetzel et al., 2014; Grossmeier et al., 2016).

#### Background/Demographic Characteristics

Four demographic variables were also used to validate the WBA. These were as follows: (1) gender (male vs. female; category “undefined,” despite being made available to respondents, was excluded from the analysis due to low frequency; only one participant chose it); and (2) age (30 or below, 31–40, 41–50, and above 50), education (some college but no degree, associate degree, bachelor's degree, and graduate school), and marital status (category of being married was distinguished).

### Statistical Analysis

#### Analytical Strategy

We intended to validate the WBA using a construct validity approach. Consequently, we examined specific features of the WBA construct, that is, its domains and structure, using empirical techniques such as factor analysis and multitrait–multimethod (MTMM) analysis. Our special focus was on the dimensionality of the WBA, as we intended to demonstrate that the instrument has consistent but distinct domains (i.e., emotional health, physical health, meaning and purpose, character strengths, social well-being, and financial security). We also examined correlations between WBA domains and external self, and we objectively reported variables as well as demographic characteristics to position the WBA in a broader conceptual space, establishing a logical and theoretically consistent pattern of relations between WBA domains and other variables.

We sought to construct a six-domain well-being instrument that demonstrates (Marsh et al., 2019) (1) good reliability: median Cronbach's alpha coefficient at least 0.80 across the scales (T1 and T2); (2) good test–retest stability over 1 year: median test–retest correlation of at least 0.70 across the six scales (repeated sample from T1 and T2); (3) a well-defined factor structure as shown by the traditional indices of fit employed in structural equation modeling (SEM) (T1 and T2); (4) satisfactory stability and factor structure generalizability based on responses from multiple time points (T1 and T2); (5) a factor structure that is invariant across gender, age groups, marital status, and educational attainment as shown by multiple-group structural equation models (T1 and T2); and (6) convergent and discriminant validity in relation to (i) MTMM analyses of WBA responses in relation to time (test–retest stability, T1 and T2), (ii) objective measures of health (e.g., diagnosis of disease derived from insurance claim data, T2), (iii) self-reported measures of health (T2), and (iv) selected demographic variables (gender, age, marital status, and education).

#### Factor Analysis

Beyond the standard data screening for missing values (only cases with complete data were included in the analysis), descriptive analyses, reliability estimates, and factor analysis were the key statistical methods used in this study.

Following theoretical arguments and practical solutions of Marsh et al. (2012, 2019), in this study, we used both confirmatory factor analysis (CFA) and exploratory SEM (ESEM) approaches for testing the psychometric properties of the scale, while test–retest stability and MTMM analyses were used to assess convergent and discriminant validity. Our reliance on both tools results from the conviction that we share with other scholars (e.g., Marsh et al., 2012, 2019) that CFAs of multidimensional constructs often fail to meet standards of good measurement in terms of goodness of fit, measurement invariance (MI), lack of differential item functioning, and well-differentiated factors in support of discriminant validity. This is usually due to the very strong assumption that each item loads on a single factor only (no cross-loadings are allowed). ESEM, which integrates the best aspects of CFA/SEM and traditional exploratory factor analysis (EFA), allows to simultaneously introduce cross-loadings (as in EFA) and conduct assessment of goodness of fit (as in CFA) (Asparouhov and Muthén, 2009; Marsh et al., 2009, 2020). Since this tool provides confirmatory tests of *a priori* factor structures, allows for evaluation of relations between latent factors, performs multigroup tests of MI (e.g., configural, metric, and scalar invariance), and leads to a better differentiation among the multiple factors, it has been proven useful in the scale development and scale validation studies (Marsh et al., 2012, 2019; Tóth-Király et al., 2017).

As the WBA was designed to comprise six distinct, yet correlated, domains of well-being, the preliminary analysis focused on examining each WBA domain independently using CFA models only (this did not affect the general approach, because one-factor models in EFA, CFA, and ESEM are equivalent). Only in the main analysis did we aim to provide evidence of the psychometric qualities of the six-domain WBA tool with items from all six domains included simultaneously in the analysis. To this end, the ESEM was used, but CFA results are also reported for comparisons.

To evaluate the goodness of fit for the factor models, we calculated the goodness-of-fit indices that are robust with respect to sample size (Hu and Bentler, 1999), including the comparative fit index (CFI), the Tucker–Lewis index (TLI), and the root-mean-square error of approximation (RMSEA). For the CFI and TLI, values >0.95 indicate a satisfactory fit, although values >0.90 are also acceptable (Hu and Bentler, 1999; Marsh et al., 2012). For RMSEA, values <0.08 indicate a satisfactorily low level of noise in the model, and below 0.06 indicate a very low level of noise (Hu and Bentler, 1999).

Robust Maximum likelihood (MLR) estimation, available in Mplus, was used for model testing. This approach provides standard errors and tests of model fit that are robust to the non-normality of the data (Yuan and Bentler, 2000). This estimator is also preferred when there are five or more response categories (Rhemtulla et al., 2012). Analyses were conducted using Stata 15 and Mplus 8.

#### Multiple Group Tests of Factorial Invariance

The MI testing included a series of model comparisons in the multiple-group factor analytical framework. Following the arguments and empirical approaches in tests of MI by Marsh et al. (2004, 2012, 2019), the fit of the multigroup configural, metric, and scalar models was examined using the fit statistics. Although a commonly accepted approach to examine changes in CFI and RMSEA (Chen, 2007) has emerged, Marsh et al. (2004, 2012, 2019) have continuingly emphasized that these cutoff values for CFI, TLI, and RMSEA constitute rough guidelines only. They should not be treated as “golden rules,” especially in the ESEM approach (Marsh et al., 2020).

The series of tests of invariance was conducted. First, we evaluated whether the factor structures for the two groups completing WBA at T1 only, or at both T1 and T2, are invariant. Second, we scrutinized invariance of the factor solution over gender, age groups, marital status, and educational categories. Since each of these grouping variables is substantively different, favorable results of tests of invariance potentially provide evidence for the generalizability and robustness of the WBA factor structure in relation to these demographic variables. The traditionally used cutoff values for fit indices were used to evaluate model fit.

#### Convergent and Discriminant Validity: Multitrait–Multimethod Analyses

The MTMM design (Campbell and Fiske, 1959) in relation to time was used to test the convergent and discriminant validity of WBA (Marsh et al., 2010a). In this framework, convergent validity is test–retest correlation (and refers to stability over time), and the different “methods” refer to time (as in the example by Marsh et al., 2010a). Based on test–retest (longitudinal) data from participants who took the survey at both T1 and T2, the MTMM analysis was conducted in a factor analytical framework, that is, using the latent correlation matrix of correlation coefficients among six WBA domains.

This latent MTMM matrix provides more accurate estimates of convergent and discriminant validity than the traditional MTMM matrix [i.e., constructed under the classical test theory framework (Novick, 1966)] as evidenced by Marsh (1993). Additionally, when conducted in relation to time, it provides the best case test of the discriminant validity of a multidimensional measure (Marsh et al., 2019).

#### Associations With Background/Demographic Characteristics

In subsequent analyses, we added demographic variables (gender, age, education, and marital status) to the WBA factor structure. In particular, the set of demographic characteristics was regressed on the set of WBA factors corresponding to the WBA domains to examine the pattern of associations and to provide further validation of the multidimensional structure of the WBA.

#### Convergent and Discriminant Validity: Correlations With Other Constructs

We also examined correlations between WBA domains and (1) objective measures of health and (2) self-reported measures of health. The examination of convergent validity was based on the analysis of correlations between WBA domains and a set of external criteria in the ESEM framework.

## Results

### Factor Structure

#### Confirmatory Factor Analysis and Exploratory Structural Equation Modeling

An initial step was to evaluate the factor structure underlying the responses to each WBA domain. Subsequently, in the main analysis, the structure of the 40-item WBA instrument was tested, and the results based on CFA and ESEM were compared. Factor analysis was conducted on the entire set of 3,579 responses from participants at T1 and T2. To benefit from all collected data, we constructed a long (stacked) file with 1,161 records from participants who took the survey at T1 only, and 2,418 records from 1,209 participants who contributed responses at both T1 and T2. For these long-format analyses, we used the Mplus complex design option to adjust standard errors for the fact that there were two sets of responses for 1,209 participants (Muthén and Muthén, 2012).

#### Factor Structure: One-Factor Models

One-factor models were evaluated with the goodness-of-fit indices. For one-factor models corresponding to one WBA domain at a time, in the case of the unsatisfactory fit of a single-group CFA model, modification indices were examined. Modifications in model specification were used with caution and applied only if a reasonable theoretical explanation existed, since the *ad hoc* inclusion of covariance between error terms should generally be avoided (Marsh et al., 2010b). Therefore, when prompted by the modification indices to include a covariance between error terms, we first carefully examined the wording of the corresponding items. Only after we were able to find convincing arguments supporting a possible common cause for correlated error terms [e.g., similar wording of the items; the same words used; and the same particular phenomena being addressed; or negative orientation of items (Finkel, 1995; Marsh et al., 2013)], we incorporated a specific covariance term. Parallelly, ESEM available in Mplus was used (Asparouhov and Muthén, 2009; Marsh et al., 2009, 2020). A similar strategy was applied to evaluate the six-factor structure of WBA.

The CFA conducted on each WBA domain revealed a relatively moderate fit of most of the domain-specific models (Table 2). The CFA provided a sufficiently good fit only for the character strengths domain (CFI = 0.962, TLI = 0.943, RMSEA = 0.067). Acceptable fit according to CFI and TLI criteria, but not according to RMSEA, was recorded also for meaning and purpose domain (CFI = 0.936, TLI = 0.904, RMSEA = 0.106) as well as for financial security (CFI = 0.940, TLI = 0.900, RMSEA = 0.130). For the remaining three domains, satisfactory model fit was observed only after the three domains were analyzed under the less restrictive and less parsimonious two-factor ESEM framework. In this framework, factor loadings on the second factor concerned the two pairs of negatively oriented items or two items related to the community well-being (exactly the same as identified by modification indices in the CFA) (details in the notes to Table 2).

**Table 2:** Goodness of fit for models based on the T1 and T2 stacked file (*n* = 3,579).

	**CFI**	**TLI**	**RMSEA [90% CI]**
**Domain specific models**			
Emotional health one-factor CFA	0.899	0.849	0.120 [0.112; 0.127]
Emotional health one-factor CFA+covariance between the error terms^a^	0.933	0.892	0.101 [0.094; 0.109]
Emotional health two-factor ESEM	0.971	0.924	0.085 [0.075; 0.095]
Physical health one-factor CFA	0.907	0.860	0.105 [0.098; 0.113]
Physical health one-factor CFA—limited item set^b^	0.968	0.947	0.068 [0.059; 0.078]
Physical health two-factor ESEM	0.983	0.956	0.059 [0.049; 0.069]
Meaning and Purpose one-factor CFA	0.936	0.904	0.106 [0.099; 0.114]
Purpose one-factor CFA+covariance between the error terms^c^	0.969	0.950	0.077 [0.069; 0.085]
Purpose two-factor ESEM	0.976	0.938	0.086 [0.076; 0.096]
Character strengths	0.962	0.943	0.067 [0.060; 0.075]
Social well-being one-factor CFA	0.865	0.798	0.151 [0.143; 0.158]
Social connectedness one-factor CFA+covariance between the error terms^d^	0.973	0.956	0.070 [0.063; 0.078]
Social connectedness two-factor ESEM	0.988	0.969	0.059 [0.049; 0.069]
Financial security one-factor CFA	0.940	0.900	0.130 [0.121; 0.140]
Financial security one-factor CFA+covariance between the error terms^e^	0.964	0.933	0.106 [0.097; 0.116]
Financial security two-factor ESEM	0.994	0.978	0.061 [0.047; 0.075]
**WBA model**			
Six-factor CFA	0.872	0.862	0.058 [0.057; 0.059]
Six-factor ESEM	0.918	0.884	0.053 [0.052; 0.054]
Six-factor ESEM+covariance of error terms^f^	0.941	0.916	0.045 [0.044; 0.046]

*CFI, comparative fit index; TLI, Tucker–Lewis index; RMSEA, root-mean-square error of approximation; CFA, confirmatory factor analysis; ESEM, exploratory structural equation modeling; WBA, Well-Being Assessment*.

a *Covariance between error terms of two negatively oriented items in the emotional health domain: depression and anxiety*.

b Without the item “I regularly do things to maintain and improve my health, in diet, exercise, and health care.”

c *Covariance between error terms of items: “My life has a clear sense of purpose” and “I understand my purpose in life” due to the similar wording and the method effect (these items were placed one after another in the questionnaire)*.

d Covariance between error terms of the only two items refereeing particularly to participant's broader community: “I feel connected to the broader community around” and “People in my broader community trust and respect one another.”

e Covariance between error terms of the only two negatively oriented items: “How often do you worry about food, housing, or health expenses?” And “The amount of debt I have often overwhelms me.”

f *Covariance between error terms of (1) two negatively oriented items in the financial security domain; (2) two negatively oriented items in the emotional health domain; and (3) the only two items referring particularly to a participant's broader community in the social connectedness domain; detailed factor loading structure with cross-loadings is presented in the Supplementary Material 2, Table A5*.

#### Factor Structure: Six-Factor Model

A highly restrictive CFA structure, in which each item could be loaded by one factor only, provided a rather poor fit (Table 2, CFI = 0.872, TLI = 0.862, RMSEA = 0.058). A fit obtained under the less restrictive ESEM specification was better (CFI = 0.918, TLI = 0.884, RMSEA = 0.053) with goodness-of-fit indices that control for parsimony (RMSEA and TLI) indicating better fit for the ESEM than the CFA. The fit of WBA six-factor ESEM was further improved (CFI = 0.941, TLI = 0.916, RMSEA = 0.045) after adding three covariance terms previously identified as eligible in the domain-specific analyses (i.e., two terms corresponded to two pairs of negatively oriented items and one to the only two items that refer to beyond self, that is, to the respondent's community).

Additionally, parameter estimates based on the well-fitting WBA ESEM demonstrated that the WBA factors are well-defined. In particular, standardized factor loadings related to emotional health ranged |λ| = 0.488–0.789 with median Me = 0.659, while the median for cross-loadings was 0.044. For the physical health, domain factor loadings ranged |λ| = 0.423–0.848 with median Me = 0.691, while the median for cross-loadings was Me = 0.012. For the meaning and purpose domain, factor loadings ranged |λ| = 0.318–0.987 with median Me = 0.598, while the median for cross-loadings was Me = 0.041. For the character strengths domain, factor loadings ranged |λ| = 0.491–0.814 with median Me = 0.697, while the median for cross-loadings was 0.047. For the social connectedness domain, factor loadings ranged |λ| = 0.274–0.971 with median Me = 0.731, while the median for cross-loadings was Me = 0.039. For the financial security domain, factor loadings ranged |λ| = 0.560–0.906 with median Me = 0.771, while the median for cross-loadings was Me = 0.029.

The pattern of loadings and cross-loadings provided sufficient support for the *a priori* factor structure relating the 40 items to the six domains of the WBA (parameter estimates are presented in the Supplementary Material 2, Table A5). Consequently, since the ESEM framework proved superior to the CFA framework for the WBA, the ESEM framework was given priority in the subsequent investigations.

#### Multiple-Group Tests of Factorial Invariance

In the first step, tests of invariance were conducted to examine whether the factor structures for the groups of respondents completing WBA at T1 and at T2 were invariant. Examination of both (1) six one-factor models corresponding to six respective domains of WBA and (2) the six-factor model of WBA was undertaken. Under the ESEM framework, we found good support for all three types of invariance: from the least restrictive configural invariance (i.e., no invariance constraints) through the less restrictive models of metric invariance (i.e., invariance of factor loadings), to the most restrictive model of scalar invariance (i.e., invariance of factor loadings and intercepts). This conclusion applied to both 6 one-factor models (detailed results presented in the Supplementary Material 1, Table A3 and Supplementary Material 2, Table A6) and the six-factor model of WBA (results in Table 3). This suggested that WBA structure comprising six domains is robust in relation to time.

**Table 3:** Goodness of fit for time measurement invariance models based on the T1 and T2 stacked file.

**Model**	**CFI**	**TLI**	**RMSEA**
**WBA six-factor ESEM+covariance of error terms**			
Configural	0.941	0.916	0.047
Metric	0.940	0.929	0.043
Scalar	0.938	0.918	0.046

*Covariance between error terms of the two negatively oriented items in the financial security domain, two negatively oriented items in the emotional health domain, and the only two items referring particularly to participant's broader community in the social connectedness domain as described in the notes to Table 2*.

*CFI, comparative fit index; TLI, Tucker–Lewis index; RMSEA, root-mean-square error of approximation; WBA, Well-Being Assessment; ESEM, exploratory structural equation modeling*.

In the second step, tests of invariance for the six-factor WBA model in relation to gender, age, marital status, and education were conducted (Table 4). Under the ESEM framework, we found favorable results of tests of invariance with respect to these four demographic characteristics. They provided evidence of the generalizability and robustness of the WBA factor structure in relation to these demographic variables.

**Table 4:** Goodness of fit for gender, age, education, and marital status measurement invariance ESEM model based on the T1 and T2 stacked file.

**Model**	**CFI**	**TLI**	**RMSEA**
**Gender invariance**			
Configural	0.937	0.911	0.047
Metric	0.940	0.928	0.043
Scalar	0.939	0.929	0.042
**Age invariance**			
Configural	0.932	0.906	0.046
Metric	0.934	0.925	0.041
Scalar	0.929	0.923	0.042
**Marital status invariance**			
Configural	0.939	0.914	0.046
Metric	0.939	0.928	0.042
Scalar	0.937	0.926	0.043
**Education invariance**			
Configural	0.935	0.908	0.048
Metric	0.937	0.929	0.042
Scalar	0.935	0.929	0.042

*Gender—male vs. female. Marital status—married vs. otherwise. Age—below 40, 41–50, and 51+. Education—at most some collage, associate degree or bachelor's degree, and graduate degree*.

*ESEM, exploratory structural equation modeling; CFI, comparative fit index; TLI, Tucker–Lewis index; RMSEA, root-mean-square error of approximation*.

### Convergent and Discriminant Validity in Relation to Time

The MTMM analyses were conducted on the panel sample (T1 and T2) with time as the method factor to provide evidence on the stability over time of six WBA domains. In the MTMM, conceptualized with time as the method factor, the convergent validities are the six test–retest correlations between matching T1 and T2 factors corresponding to the WBA domains (bolded and underlined correlations in Table 5). These were all above 0.7 (0.731–0.873; Me = 0.760; mean = 0.783), which provided strong support for convergent validity in relation to time as the method factor.

**Table 5:** Test–retest latent correlations among six Well-Being assessment domains: a multitrait–multimethod (MTMM) latent matrix.

	**F1–T1**	**F2–T1**	**F3–T1**	**F4–T1**	**F5–T1**	**F6–T1**	**F1–T2**	**F2–T2**	**F3–T2**	**F4–T2**	**F5–T2**	**F6–T2**
Emotional health (F1–T1)	1											
Physical health (F2–T1)	0.599	1										
Purpose (F3–T1)	0.769	0.446	1									
Character strengths (F4–T1)	0.740	0.472	0.716	1								
Social connectedness (F5–T1)	0.604	0.412	0.668	0.633	1							
Financial security (F6–T1)	0.495	0.467	0.378	0.393	0.253	1						
Emotional health (F1–T2)	**0.767**	0.505	0.624	0.607	0.488	0.449	1					
Physical health (F2–T2)	0.522	**0.826**	0.395	0.411	0.311	0.439	0.640	1				
Purpose (F3–T2)	0.576	0.392	**0.731**	0.561	0.503	0.317	0.796	0.501	1			
Character strengths (F4–T2)	0.589	0.397	0.581	**0.749**	0.474	0.356	0.788	0.517	0.726	1		
Social connectedness (F5–T2)	0.498	0.391	0.554	0.488	**0.753**	0.261	0.617	0.468	0.716	0.657	1	
Financial security (F6–T2)	0.421	0.388	0.333	0.354	0.204	**0.873**	0.518	0.453	0.376	0.455	0.309	1

*Bolded and underlined numbers are test–retest correlations. Gray cells in the upper five rows present correlation coefficients among the six WBA domains at T1. Gray cells in the lower five rows present correlation coefficients among the six WBA domains at T2. All correlation coefficients are significant at p < 0.001*.

*WBA, Well-Being Assessment*.

Evidence for discriminant validity are to be found off the diagonal because measures of different constructs should not correlate highly with each other. If off-diagonal correlations are uniformly lower than the convergent coefficients (bolded and underlined correlations in Table 5), these lower correlations together with higher convergence coefficients constitute evidence for validity.

We found that the median correlation among the six distinct WBA domains at T1 was 0.495 (gray cells in the upper five rows of Table 4; 0.253–0.769; mean = 0.536), the median correlation among the six distinct WBA domains at T2 was 0.518 (gray cells in the lower five rows of Table 5; 0.309–0.796; mean = 0.569), and the median correlation between these six distinct domains at T1 and T2 was 0.444 (0.204–0.624; mean = 0.446). Since each convergent validity is substantially greater than the mean of off-diagonal correlations, there is strong support for both convergent and discriminant validity of all six WBA domains in relation to time. In summary, even though some of the WBA domains are substantively correlated, based on this MTMM analysis, clear evidence was found that all the WBA domains are well-differentiated.

### Convergent and Discriminant Validity in Relation to Other Constructs

Convergent validity assessment was further assessed based on the analysis of correlations between WBA domains and a set of external criteria in the ESEM framework. In particular, six latent WBA domains were correlated with eight external variables. Consequently, 48 correlations coefficients were examined for the analysis of convergent and discriminant validity (Table 6).

**Table 6:** Correlations of WBA domains with external variables.

**External variable**	**Emotional**	**Physical**	**Purpose**	**Character**	**Social**	**Financial**
	**health**	**health**		**strengths**	**connectedness**	**security**
Diagnosis of depression	−0.330^***^	−0.251^***^	−0.241^***^	−0.126^**^	−0.210^***^	−0.215^***^
Diagnosis of anxiety	−0.212^***^	−0.151^***^	−0.133^***^	−0.062	−0.095^**^	−0.106^***^
Diagnosis of migraines and other headaches	−0.066^*^	−0.161^***^	−0.046	−0.019	−0.052	−0.111^***^
Diagnosis of obesity	−0.043	−0.163^***^	−0.004	−0.004	−0.005	−0.0114^***^
Negative affect (feeling down, depressed, and hopeless)	−0.435^***^	−0.290^***^	−0.316^***^	−0.174^***^	−0.314^***^	−0.217^***^
Stress level	−0.453^***^	−0.284^***^	−0.284^***^	−0.241^***^	−0.302^***^	−0.285^***^
Number hours of sleep	0.182^***^	0.135^***^	0.131^***^	0.115^***^	0.167^***^	0.155^***^
General health	0.323^***^	0.545^***^	0.234^***^	0.231^***^	0.235^***^	0.349^***^

*WBA, Well-Being Assessment*.

*** *p < 0.001*;

** *p < 0.01*;

* *p < 0.05*.

Objective measures of mental health, that is, a diagnosis of depression and a diagnosis of anxiety, were found to be negatively associated with both health-related WBA domains. Additionally, they were also negatively associated with remaining WBA domains (with the exception of anxiety diagnosis that was not associated with character strengths domain). Regarding the objective measures of physical health, diagnosis of migraines and other headaches was negatively associated with emotional health, physical health, and financial security, while diagnosis of obesity was found to be negatively associated with the physical health domain and additionally with the financial security domain. Regarding the self-reported measures of health (i.e., general health, number of sleep hours, stress level, and negative affect), stress level and negative affect were associated negatively, while general health and number of sleep hours were associated positively with each of the WBA domains.

### Classical Test Reliability and Test–Retest Correlations

Reliability of the WBA domains and WBA itself was examined under the classical test theory framework (Novick, 1966) (Table 7). Cronbach's alpha was computed 2-fold: on responses collected at T1 and at T2. All coefficients were above 0.86 with the median alpha at T1 amounting to Me = 0.88 and at T2 amounting to Me = 0.91. The median test–retest correlation was 0.71 (0.666–0.833). These findings indicated satisfactory reliability of each of the WBA domains as well as the six-domain WBA instrument itself.

**Table 7:** Reliability—Cronbach's alpha and test–retest reliability.

	**Cronbach's alpha at T1**	**Cronbach's alpha at T2**	**Test–retest reliability**
Emotional health	0.867	0.873	0.713^***^
Physical health	0.869	0.891	0.748^***^
Purpose	0.925	0.945	0.714^***^
Character strengths	0.866	0.899	0.666^***^
Social connectedness	0.901	0.917	0.709^***^
Financial security	0.911	0.930	0.833^***^
WBA	0.951	0.958	0.795^***^

*WBA, Well-Being Assessment*.

*** *p < 0.001*.

### Associations With Background/Demographic Characteristics

We found different patterns of associations between the six domains of WBA and four demographic variables examined (Table 8). Out of 54 estimates of the six estimated multiple indicators, multiple causes (MIMIC) models, 19 were statistically significant. Although this complex pattern of associations is important in its own right, it additionally provided solid evidence supporting our approach to the WBA as a tool covering a multidimensional structure. In particular, we found evidence that complex multidimensional patterns of associations could not have been represented with a single global measure of well-being.

**Table 8:** Association between six WBA factors and demographic variables.

**Predictor variables**	**Emotional**	**Physical**	**Meaning and**	**Character**	**Social**	**Financial**
	**health**	**health**	**purpose**	**strengths**	**connectedness**	**security**
**Male**	0.068	0.022	−0.085	−0.175^**^	−0.187^**^	0.133^*^
**Married**	0.213^***^	0.187^***^	0.247^***^	−0.008	0.261^***^	0.240^***^
**Age group (ref**. **=** **30 or below)**						
31–40	0.074^*^	−0.124	0.118	0.108	−0.085	−0.205^**^
41–50	0.041	−0.256^***^	0.107	0.108	−0.178^*^	−0.122^*^
Above 50	0.322^***^	−0.066	0.244^**^	0.206^**^	−0.049	0.335^***^
**Education (ref**. **=** **high school diploma or equivalent**						
Some college but no degree	0.025	−0.021	−0.053	−0.017	−0.053	−0.090
Associate degree	−0.032	0.047	−0.034	−0.076	−0.066	0.043
Bachelor's degree	0.064	0.211^*^	−0.065	−0.155	−0.100	0.356^***^
Graduate school	0.205	0.283^**^	0.058	−0.077	−0.027	0.552^***^

*WBA 6-factor ESEM model with covariates was estimated (MIMIC model). Each WBA domain was represented by a latent factor and regressed on the four demographic covariates*;

*** *p < 0.001*;

** *p < 0.01*;

* *p < 0.05*.

*WBA, Well-Being Assessment; ESEM, exploratory structural equation modeling; MIMIC, multiple indicators, multiple causes*.

Evaluating the associations between the WBA domains and major demographic variables, we found that males reported higher financial security than females, while females reported more character strengths and higher social connectedness than males. Being married was positively related to five out of six WBA domains, with the largest associations observed with social connectedness, meaning and purpose, and financial security (effect size of ~0.25). Being married was, however, not associated with character strengths. Also, in support of the multidimensional structure, we noted that the oldest participants scored higher than younger ones in the domains of meaning and purpose, emotional health, and character strengths. For the remaining WBA domains, however, the pattern was more complex. For example, while the oldest participants also reported higher financial security, those in the 31–50 age group reported the lowest financial security. Although most participants in different age groups did not differ in reporting social connectedness and physical health, those in the 41–50 age group reported significantly lower scores for these two WBA domains. As for emotional health, the highest self-reports were provided by the oldest participants, followed by those between 31 and 40. Finally, although most WBA domains were not associated with education, a clear pattern of higher financial security among better educated respondents was observed. In addition, participants with a bachelor's degree reported significantly higher physical health than others.

## Discussion

We aimed to develop a robust multidimensional measure of complete well-being based on the conceptual model of human flourishing developed by VanderWeele (2017) and VanderWeele et al. (2019), the WHO definition of health, and a broader review of the well-being and health literature. By defining well-being within the framework of complete well-being, or human flourishing, understood as “a state in which all aspects of a person's life are good” (VanderWeele, 2017, p. 8149), our approach extends the well-being measurement literature by including domains beyond psychological well-being.

Findings of the current study indicated that the six-factor model underlying the WBA is broadly supported by the CFA and ESEM. Consequently, the WBA six-domain comprehensive instrument that we proposed can be used to assess holistic well-being in terms of its six domains: emotional health, physical health, meaning and purpose, character strengths, social connectedness, and financial security. Although each of these domains is distinct, all of them are nearly universally desired, and all but financial security constitute ends in themselves (VanderWeele, 2017; Weziak-Bialowolska et al., 2019). The six WBA domains showed good reliability, test–retest correlation, convergent/discriminant validity in relation to stability over time and relevant health measures, and a good fit to the data that was invariant over time, and across gender, age, education, and marital status.

The results of the current study support the conclusions of Hone et al. (2014) that flourishing measures are built on a foundation of positive social relationships, positive affect, and purpose or meaning in life. Our results add to the literature by providing evidence that the character domain can be incorporated in the well-being measurement. So far, this domain was either included indirectly in flourishing instruments (see for example Su et al., 2014) or measured as an unrelated phenomenon (see for example McGrath, 2015; Blanchard et al., 2019). However, the importance of character strengths and virtues for human flourishing has been long argued by philosophers (Pieper, 1966; Aristotle and Brown, 2009; Baril, 2016) and, more recently, also by psychologists (Graziosi et al., 2020; Niemiec, 2020; Weziak-Bialowolska et al., 2021). Additionally, this study corroborates that, although measures of mental state are covered by instruments of psychological well-being (Ryff and Keyes, 1995) and measures of flourishing are covered by instruments of psychological and social well-being (Diener et al., 2010), a more holistic conceptualization of flourishing goes beyond these areas of human functioning. In particular, this study provides evidence that physical health is just as essential quality for personal well-being as is emotional well-being, confirming theoretical arguments of VanderWeele et al. (2019) and prior findings of Weziak-Bialowolska et al. (2019). This is also reflected in research on the importance of the individual well-being domains, which found physical health to be the top-ranked domain (Lee et al., 2021a). Finally, the results of this study also indicate that, if the aim is to measure complete human well-being, qualities allowing for the persistence of well-being should be included in measurement. Theoretical arguments imply that financial conditions belong to such qualities (VanderWeele, 2017) and empirical evidence on the good fit of the six-factor model underlying the WBA with financial security domain included provides further confirmation.

Despite these positive findings, our study also has some limitations. We note, first, that although our approach to examine the factor structure of the WBA is in line with growing body of research on ESEM (Marsh et al., 2012, 2019, 2020; Tóth-Király et al., 2017), we also observed some reluctance with respect to this approach in the research community. Nonetheless, we followed the ESEM logic because it has been theoretically and empirically documented that it results in a more realistic representation of covariance structure than its traditional counterpart—CFA (Asparouhov and Muthén, 2009; Marsh et al., 2009).

Second, two non-zero cross-loadings were observed that could have challenged the theoretical model. Although the cross-loadings were present, they did not undermine the meaning of the WBA domains. However, two of them (referring to one item of the meaning and purpose domain: “Overall, to what extent do you feel the things you do in your life are worthwhile?” And one item of the social well-being domain: “How often do you feel lonely?”) had a substantial presence in the emotional health domain, which seemed plausible. The former item is a well-known indicator of eudaimonic psychological well-being (Ryff and Singer, 2008) that reflects the experience of existence and refers to overall relatedness and sense of one's experiences (King et al., 2006). Additionally, in prior factor analytical approaches, it has been already evidenced to be grouped together with other indicators of emotional well-being (Weziak-Bialowolska et al., 2019). The latter item, instead, can be understood as an indicator of not only social but also emotional isolation (Weiss, 1973; Perlman and Peplau, 1981; Beutel et al., 2017). Consequently, the overall pattern of loadings and cross-loadings reflected the theoretical structure that comprises the six distinct domains of the WBA.

Third, despite using the less restrictive framework of ESEM (compared with CFA), three pairs of correlated errors were included in the final measurement model. As suggested by the literature (Marsh et al., 2010b), this decision was made only after convincing arguments supporting a possible common cause for correlated error terms were identified. In the first two cases, they concerned pairs of negatively oriented items. It is worth noting that the 40-item WBA instrument includes only five negatively oriented items. Two of them are in the emotional health domain, two in the financial security domain, and one in the physical health domain. Those pairs in the emotional health and the financial security domains required correlated error terms, which might have been an artifact resulting from the method factor (Finkel, 1995; Marsh et al., 2013). In the remaining case, the correlated error term corresponded to the only two items in which a collective perspective was adopted (i.e., two items related to communal social well-being in the social connectedness domain) as opposed to the distinctly individual nature of the remaining 38 items. Although we believe that our explanations for the three correlated error terms are plausible and provide sufficient justification for the inclusion of these terms, we recognize that there might be some minor issues that might warrant a reexamination of these items. For instance, the reorientation of negatively oriented items through an adjustment of their wording (e.g., by using antonyms) may be considered. Finally, since the sample of U.S. working adults was used to establish psychometric properties of the WBA instrument, further research is needed to establish generalizability of the WBA instrument with a community sample and with other nationalities, especially from non-Western, culturally different populations.

With psychometric properties well-established, this new measure of well-being may be used more broadly. For example, the WBA can advance research and policies related to health and well-being. The instrument may be considered for use by scientists and practitioners in psychology and related social sciences, as well as clinicians, public health officials, and patients. These three latter groups often equate the absence of a disease with health, applying a deficit-reduction framework to health (VanderWeele et al., 2019) and disregarding other outcomes perceived by people as central for well-being (e.g., happiness and social connections). Adoption of more holistic measures of well-being that go beyond psychological well-being and consider a positive health approach may help to shift the focus of clinicians and public health officials from only the alleviation of health deficiencies toward health and well-being promotion. For patients facing serious treatment decisions (e.g., men wrestling with treatment decisions over relatively advanced stage bladder cancer, knowing that a cystectomy will maximize life expectancy but severely hamper quality of life and happiness), asking questions related to distinct domains of complete well-being can inform complex trade-offs (VanderWeele et al., 2019). Future research in groups of patients with specific diseases will strengthen the applicability of the WBA.

## Application of the Well-Being Assessment

This 40-item instrument can be used to assess well-being in six domains: (1) emotional health (e.g., “How satisfied are you with life as a whole these days?”); (2) physical health (e.g., “How would you rate your physical health?”); (3) meaning and purpose (e.g., “I have values and beliefs that help me understand who I am.”); (4) character strengths (e.g., “I always act to promote good in all circumstances, even in difficult and challenging situations.”); (5) social connectedness (e.g., “My relationships are as satisfying as I would want them to be.”); and (6) financial security (e.g., “I am able to meet my normal monthly living expenses without any difficulty.”). The responses to each of the 40 items are measured on an 11-point Likert scale ranging from 0 to 10 (details in Table 1). A domain-specific score can be calculated for each domain by averaging the responses across all items included in the domain. Since some items are negatively oriented (denoted with *r* in Table 1), reverse coding is necessary to ensure that a higher score indicates greater well-being.

The overall WBA score can be calculated by simply averaging the composite scores across all six domains. Lack of weights in the WBA computation formula implies equal importance of all domains. This approach is supported by the empirical evidence on the comparable importance of each of the domains (Lee et al., 2021a).

Because it includes neglected domains of physical health, character, and virtue and the enabling domain of financial security, the WBA offers benefits for researchers interested in the assessment of complete well-being beyond measures that focus on emotional well-being only. We recommend using the full WBA instrument for an overall assessment of complete well-being for virtually all people because the domains are nearly universally valued (VanderWeele, 2017; Lee et al., 2021a). The WBA is also useful for identifying specific of domains of excellence for individuals and groups. However, if researchers are interested in particular domains of the WBA (e.g., character strengths) or are unable to include the entire 40-item WBA, we recommend using a subset of items corresponding to the domain of interest. Since our analyses provided support for satisfactory psychometric properties and robustness of not only the WBA instrument but also each of its domains, measures of single well-being domains can be represented as a single total score.

## Data Availability Statement

The raw data supporting the conclusions of this article will be made available by the authors, without undue reservation.

## Ethics Statement

The studies involving human participants were reviewed and approved by Harvard T.H. Chan School of Public Health Institutional Review Board. The patients/participants provided their written informed consent to participate in this study.

## Author Contributions

DW-B contributed to the study concept, data analysis, and interpretation of the result, she also drafted and revised the manuscript. PB contributed to data analysis and interpretation of the result, he also revised the manuscript. ML and YC contributed to interpretation of the results and revised the manuscript. TV developed the study design and the study concept, contributed to interpretation of the results, revised the manuscript, and provided funding for the study. EM developed the study design and provided funding for the study. All authors approved the final version of the manuscript.

## Conflict of Interest

EM and TV report receiving grants and personal fees from Aetna Inc. EM reports receiving grants from the Levi Strauss Foundation; she also reports serving as director of SHINE at Harvard (Sustainability and Health Initiative for NetPositive Enterprise); support is made possible through SHINE from multiple companies. TV reports receiving grants from the John Templeton and personal fees from Flerish Inc. and Flourishing Metrics. The research findings represent the perspective of the authors and do not reflect the opinions or endorsement of any organization.

## Footnotes

^1^https://www.who.int/about/mission/en/. Accessed June 3, 2020.
