# Supplementary material for: Psychometric Properties of Flourishing Scales From a Comprehensive Well-Being Assessment
Source: Front Psychol. 2021 Apr 21;12:652209. doi: 10.3389/fpsyg.2021.652209 (PMC8097094; doi:10.3389/fpsyg.2021.652209)
Supplement: Supplementary file 1 [file Table_1.docx]

Supplementary Material

**Supplementary Material 1.** Preliminary analysis based on data collected at time T0 (n=276).

**Table A1. Domain-specific models - factor loadings and reliability of each WBA domain at T0**

| **Domain and items** | Factor1 | Alpha |
| --- | --- | --- |
| **Emotional Health** |  | 0.869 |
| EH1 | 0.7571 |  |
| EH2 | 0.7749 |  |
| EH3 | 0.6219 |  |
| EH4 | 0.8107 |  |
| EH5 | -0.7529 |  |
| EH6 | -0.5958 |  |
| *EH8* | *0.4957* |  |
| *EH9* | *0.7005* |  |
| EH7 | 0.604 |  |
| **Physical health** |  | 0.891 |
| PH1 | 0.6165 |  |
| PH2 | 0.7604 |  |
| PH3 | 0.7179 |  |
| *PH8* | *0.737* |  |
| PH4 | 0.8755 |  |
| PH5 | -0.5926 |  |
| PH6 | 0.8836 |  |
| PH7 | 0.6037 |  |
| **Meaning and purpose** |  | 0.903 |
| MP1 | 0.7885 |  |
| MP2 | 0.8036 |  |
| MP3 | 0.9224 |  |
| MP4 | 0.8852 |  |
| MP5 | 0.6976 |  |
| *MP7* | *0.7375* |  |
| MP6 | 0.3559 |  |
| *MP8* | *0.7704* |  |
| **Social connectedness** |  | 0.902 |
| SC1 | 0.7814 |  |
| SC2 | 0.5288 |  |
| SC3 | -0.3167 |  |
| SC4 | 0.7899 |  |
| SC5 | 0.309 |  |
| SC6 | 0.0791 |  |
| SC7 | 0.0479 |  |
| **Character strengths** |  | 0.840 |
| CS1 | 0.7045 |  |
| CS2 | 0.6315 |  |
| CS3 | 0.7017 |  |
| CS4 | 0.7256 |  |
| CS5 | 0.7553 |  |
| CS6 | 0.6234 |  |
| **Financial security** |  | 0.940 |
| FS1 | 0.8199 |  |
| FS2 | -0.7442 |  |
| FS3 | 0.8543 |  |
| FS4 | 0.9122 |  |
| FS5 | 0.8376 |  |
| *FS7* | *0.8933* |  |
| FS6 | -0.8132 |  |

*Note:* Domain-specific exploratory factor analysis was conducted. Item codes correspond to item wording presented in Table1 in the manuscript. Item codes and factor loadings corresponding to six items discarded after T0 are underlined and cursive.

**Table A2. WBA Six-Factor Structure: Exploratory Structural Equation Model (ESEM) at T0 (45 items)**

|  | | Emotional Health | | Meaning and Purpose | | Social Connectedness | | Financial Security | | Character Strengths | | Physical Health | |  |
| --- | --- | --- | --- | --- | --- | --- | --- | --- | --- | --- | --- | --- | --- | --- |
| Factor loadings and cross-loadings | | | | | | | | | | | | | | |
| EH1 | | **0.694*** | | 0.062 | | 0.097 | | 0.083 | | -0.064 | | 0.063 | |  |
| EH2 | | **0.818*** | | -0.055 | | 0.195 | | -0.004 | | -0.071 | | -0.020 | |  |
| EH3 | | **0.507*** | | 0.011 | | 0.061 | | -0.063 | | 0.223 | | 0.027 | |  |
| EH4 | | **0.565*** | | 0.095 | | 0.045 | | 0.047 | | 0.232* | | 0.029 | |  |
| EH5 | | **-0.491*** | | -0.02 | | -0.215 | | -0.044 | | -0.028 | | -0.072 | |  |
| EH6 | | -0.312 | | -0.055 | | -0.267 | | -0.049 | | 0.026 | | -0.038 | |  |
| *EH8* | | **0.46*** | | 0.089 | | -0.054 | | -0.114 | | 0.116 | | -0.004 | |  |
| *EH9* | | **0.468*** | | 0.170 | | -0.027 | | -0.032 | | 0.304* | | -0.052 | |  |
| EH7 | | **0.393*** | | 0.160 | | -0.137 | | -0.008 | | 0.388* | | -0.032 | |  |
| PH1 | | 0.327* | | -0.073 | | -0.027 | | 0.156* | | 0.108 | | **0.351*** | |  |
| PH2 | | -0.168 | | 0.068 | | -0.052 | | 0.091 | | -0.038 | | **0.837*** | |  |
| PH3 | | -0.088 | | 0.024 | | -0.001 | | 0.044 | | 0.099 | | **0.723*** | |  |
| *PH8* | | 0.098 | | -0.081 | | 0.003 | | -0.065 | | 0.099 | | **0.701*** | |  |
| PH4 | | 0.076 | | -0.043 | | 0.017 | | -0.046 | | 0.008 | | **0.871*** | |  |
| PH5 | | -0.098 | | -0.079 | | -0.085 | | 0.032 | | 0.156 | | **-0.573*** | |  |
| PH6 | | -0.009 | | 0.080 | | 0.039 | | 0.002 | | -0.008 | | **0.869*** | |  |
| PH7 | | 0.128 | | -0.012 | | -0.062 | | 0.123 | | 0.163 | | **0.420*** | |  |
| MP1 | | 0.108 | | **0.588*** | | 0.092 | | -0.048 | | 0.136 | | 0.030 | |  |
| MP2 | | 0.011 | | **0.684*** | | 0.068 | | -0.022 | | 0.124 | | 0.041 | |  |
| MP3 | | 0.082 | | **0.928*** | | 0.004 | | 0.024 | | -0.053 | | 0.006 | |  |
| MP4 | | 0.052 | | **0.876*** | | -0.017 | | 0.076 | | 0.036 | | -0.003 | |  |
| MP5 | | 0.532* | | **0.272*** | | 0.028 | | 0.034 | | -0.085 | | 0.087 | |  |
| *MP7* | | 0.561* | | **0.269*** | | -0.003 | | 0.039 | | 0.083 | | -0.011 | |  |
| MP6 | | -0.184 | | **0.319*** | | 0.189 | | -0.049 | | 0.065 | | 0.165 | |  |
| *MP8* | | 0.386* | | **0.417*** | | 0.029 | | 0.014 | | 0.017 | | 0.062 | |  |
| SC1 | | 0.110 | | 0.017 | | **0.718*** | | -0.009 | | 0.044 | | 0.036 | |  |
| SC2 | | 0.008 | | 0.017 | | **0.656*** | | 0.015 | | 0.053 | | 0.027 | |  |
| SC3 | | -0.156 | | -0.116 | | **-0.441*** | | -0.043 | | 0.037 | | -0.041 | |  |
| SC4 | | 0.046 | | -0.064 | | **0.903*** | | -0.044 | | -0.01 | | 0.018 | |  |
| SC5 | | 0.007 | | -0.013 | | **0.849*** | | 0.022 | | -0.017 | | -0.002 | |  |
| SC6 | | -0.026 | | 0.181 | | **0.715*** | | 0.046 | | 0.076 | | -0.085 | |  |
| SC7 | | 0.015 | | 0.165 | | **0.611*** | | 0.01 | | 0.103 | | -0.099 | |  |
| CS1 | | 0.064 | | 0.12 | | -0.01 | | -0.026 | | **0.652*** | | -0.010 | |  |
| CS2 | | -0.013 | | 0.166 | | 0.081 | | 0.042 | | **0.503*** | | -0.011 | |  |
| CS3 | | -0.029 | | 0.049 | | 0.031 | | -0.103 | | **0.668*** | | 0.080 | |  |
| CS4 | | 0.035 | | -0.141 | | 0.073 | | 0.069 | | **0.685*** | | 0.109 | |  |
| CS5 | | 0.008 | | 0.029 | | 0.161 | | 0.140* | | **0.635*** | | 0.060 | |  |
| CS6 | | -0.058 | | -0.014 | | 0.179 | | 0.009 | | **0.55*** | | -0.012 | |  |
| FS1 | | 0.183* | | -0.125 | | 0.010 | | **0.748*** | | 0.071 | | 0.028 | |  |
| FS2 | | -0.048 | | -0.038 | | -0.077 | | **-0.655*** | | 0.092 | | -0.090 | |  |
| FS3 | | -0.034 | | -0.053 | | -0.044 | | **0.93*** | | 0.065 | | -0.078 | |  |
| FS4 | | 0.106 | | 0.048 | | 0.039 | | **0.863*** | | -0.053 | | 0.025 | |  |
| FS5 | | -0.014 | | 0.049 | | -0.009 | | **0.830*** | | 0.024 | | 0.021 | |  |
| *FS7* | | -0.008 | | 0.030 | | -0.025 | | **0.914*** | | 0.033 | | -0.049 | |  |
| FS6 | | 0.022 | | -0.006 | | -0.108 | | **-0.771*** | | 0.086 | | -0.016 | |  |
| Factor correlations | | | | | | | | | | | | | | |
|  | Emotional Health | | Meaning and Purpose | | Social Connectedness | | Financial Security | | Character Strengths | | Physical Health | |  |  |
| Emotional Health | 1 | |  | |  | |  | |  | |  | |  |  |
| Meaning and Purpose | 0.589* | | 1 | |  | |  | |  | |  | |  |  |
| Social Connectedness | 0.505* | | 0.457* | | 1 | |  | |  | |  | |  |  |
| Financial Security | 0.354* | | 0.185* | | 0.317* | | 1 | |  | |  | |  |  |
| Character Strengths | 0.238* | | 0.435* | | 0.390* | | 0.143* | | 1 | |  | |  |  |
| Physical Health | 0.466* | | 0.319* | | 0.297* | | 0.285* | | 0.280* | | 1 | |  |  |

Note: * indicates the estimates significant at at least p<0.05. Item codes correspond to item wording presented in Table1 in the manuscript. Fit statistics are: RMSEA = 0.075; CFI = 0.836; TLI = 0.779. Six items discarded after T0 are underlined and cursive.

**Table A3. *WBA Six-Factor Structure: Exploratory Structural Equation Model (ESEM) at T0 (39 items)***

|  | Meaning and Purpose | Social Connectedness | Character Strengths | Financial Security | Physical Health | (Poor) Emotional Health |
| --- | --- | --- | --- | --- | --- | --- |
| Factor loadings | | | | | | |
| EH1 | **0.363*** | 0.185 | -0.154 | 0.142* | 0.174* | -0.229 |
| EH2 | 0.258* | 0.265* | -0.158 | 0.076 | 0.095 | **-0.364*** |
| EH3 | 0.234 | 0.139 | 0.110 | -0.038 | 0.123 | **-0.199** |
| EH4 | 0.273* | -0.015 | 0.200 | 0.057 | 0.035 | **-0.514*** |
| EH5 | 0.012 | -0.002 | -0.101 | 0.000 | 0.021 | **0.921*** |
| EH6 | -0.022 | -0.083 | -0.040 | -0.021 | 0.053 | **0.670*** |
| EH7 | 0.349* | -0.105 | 0.299* | 0.004 | 0.016 | -0.194 |
| PH1 | 0.105 | 0.023 | 0.049 | 0.187* | **0.403*** | -0.09 |
| PH2 | -0.021 | -0.082 | 0.017 | 0.054 | **0.792*** | 0.032 |
| PH3 | -0.039 | -0.036 | 0.144 | 0.025 | **0.671*** | -0.039 |
| PH4 | -0.015 | 0.034 | 0.016 | -0.037 | **0.875*** | -0.021 |
| PH5 | 0.005 | 0.042 | 0.073 | 0.076 | **-0.504*** | 0.382* |
| PH6 | 0.068 | 0.046 | 0.010 | -0.02 | **0.893*** | 0.02 |
| PH7 | 0.109 | 0.011 | 0.114 | 0.136 | **0.475*** | 0.097 |
| MP1 | **0.647*** | 0.063 | 0.137 | -0.083 | 0.010 | -0.09 |
| MP2 | **0.727*** | 0.046 | 0.13 | -0.064 | 0.015 | 0.01 |
| MP3 | **0.964*** | -0.025 | -0.019 | -0.018 | -0.027 | -0.032 |
| MP4 | **0.975*** | -0.039 | 0.042 | 0.041 | -0.034 | 0.051 |
| MP5 | **0.486*** | 0.102 | -0.142 | 0.06 | 0.175* | -0.166 |
| MP6 | **0.241*** | 0.119 | 0.131 | -0.092 | 0.089 | 0.011 |
| SC1 | 0.070 | **0.738*** | 0.022 | 0.010 | 0.073 | -0.009 |
| SC2 | -0.003 | **0.650*** | 0.047 | 0.003 | 0.030 | -0.046 |
| SC3 | -0.049 | **-0.282*** | -0.035 | 0.003 | 0.037 | 0.494* |
| SC4 | -0.069 | **0.873*** | -0.01 | -0.041 | 0.025 | -0.093 |
| SC5 | -0.040 | **0.838*** | -0.02 | 0.005 | -0.001 | -0.067 |
| SC6 | 0.187* | **0.738*** | 0.061 | 0.034 | -0.071 | 0.059 |
| SC7 | 0.169 | **0.641*** | 0.091 | 0.003 | -0.077 | 0.033 |
| CS1 | 0.174 | -0.015 | **0.638*** | -0.026 | -0.012 | -0.036 |
| CS2 | 0.192 | 0.049 | **0.515*** | 0.032 | -0.033 | -0.018 |
| CS3 | 0.053 | 0.006 | **0.675*** | -0.123 | 0.068 | -0.032 |
| CS4 | -0.110 | 0.076 | **0.688*** | 0.075 | 0.11 | -0.022 |
| CS5 | 0.063 | 0.155 | **0.629*** | 0.139* | 0.059 | 0.004 |
| CS6 | -0.023 | 0.173 | **0.563*** | 0.021 | -0.024 | 0.040 |
| FS1 | -0.032 | 0.04 | 0.049 | **0.762*** | 0.065 | -0.032 |
| FS2 | -0.016 | -0.023 | 0.05 | **-0.65*** | -0.069 | 0.118 |
| FS3 | -0.032 | -0.047 | 0.075 | **0.94*** | -0.089 | 0.070 |
| FS4 | 0.119* | 0.048 | -0.057 | **0.895*** | 0.034 | 0.017 |
| FS5 | 0.041 | -0.05 | 0.051 | **0.811*** | 0.003 | -0.042 |
| FS6 | 0.036 | -0.026 | 0.027 | **-0.760*** | 0.029 | 0.120 |
| Factor correlations | | | | | | |
|  | Meaning and Purpose | Social Connectedness | Character Strengths | Financial Security | Physical Health | (Poor) Emotional Health |
| Meaning and Purpose | 1 |  |  |  |  |  |
| Social Connectedness | 0.504* | 1 |  |  |  |  |
| Character Strengths | 0.395* | 0.385* | 1 |  |  |  |
| Financial Security | 0.244* | 0.322* | 0.125 | 1 |  |  |
| Physical Health | 0.373* | 0.279* | 0.254* | 0.303* | 1 |  |
| (Poor) Emotional Health | -0.500* | -0.485* | -0.175* | -0.357* | -0.404* | 1 |

Note: * indicates the estimates significant at at least p<0.05. Item codes correspond to item wording presented in Table1 in the manuscript. Fit statistics are: RMSEA = 0.077; CFI = 0.852; TLI = 0.790.

**Supplementary Material 2.** Primary analyses based on data collected at time T1 and T2.

**Table A4. *Correlation coefficients between 40 WBA items and the WBA at T1.***

|  | EH1 | EH2 | EH3 | EH4 | EH5 | EH6 | EH7 | PH1 | PH2 | PH3 | PH4 | PH5 | PH6 | PH7 |
| --- | --- | --- | --- | --- | --- | --- | --- | --- | --- | --- | --- | --- | --- | --- |
| EH1 | 1.00 |  |  |  |  |  |  |  |  |  |  |  |  |  |
| EH2 | 0.22 | 1.00 |  |  |  |  |  |  |  |  |  |  |  |  |
| EH3 | 0.08 | 0.24 | 1.00 |  |  |  |  |  |  |  |  |  |  |  |
| EH4 | 0.00 | 0.27 | 0.19 | 1.00 |  |  |  |  |  |  |  |  |  |  |
| EH5 | 0.12 | 0.18 | 0.19 | 0.21 | 1.00 |  |  |  |  |  |  |  |  |  |
| EH6 | 0.13 | 0.19 | 0.17 | 0.15 | 0.72 | 1.00 |  |  |  |  |  |  |  |  |
| EH7 | 0.00 | 0.22 | 0.18 | 0.34 | 0.18 | 0.20 | 1.00 |  |  |  |  |  |  |  |
| PH1 | 0.06 | 0.21 | 0.15 | 0.14 | 0.14 | 0.15 | 0.18 | 1.00 |  |  |  |  |  |  |
| PH2 | 0.17 | 0.23 | 0.14 | 0.20 | 0.62 | 0.67 | 0.16 | 0.13 | 1.00 |  |  |  |  |  |
| PH3 | 0.16 | 0.19 | 0.25 | 0.12 | 0.60 | 0.62 | 0.20 | 0.12 | 0.58 | 1.00 |  |  |  |  |
| PH4 | 0.11 | 0.13 | 0.22 | 0.17 | 0.62 | 0.65 | 0.17 | 0.10 | 0.61 | 0.64 | 1.00 |  |  |  |
| PH5 | 0.11 | 0.20 | 0.18 | 0.24 | 0.75 | 0.69 | 0.22 | 0.13 | 0.67 | 0.60 | 0.65 | 1.00 |  |  |
| PH6 | 0.12 | 0.18 | 0.16 | 0.18 | 0.68 | 0.74 | 0.18 | 0.11 | 0.67 | 0.62 | 0.64 | 0.68 | 1.00 |  |
| PH7 | 0.11 | 0.17 | 0.19 | 0.17 | 0.67 | 0.70 | 0.17 | 0.16 | 0.60 | 0.58 | 0.63 | 0.64 | 0.69 | 1.00 |
| MP1 | 0.08 | 0.25 | 0.26 | 0.20 | 0.28 | 0.31 | 0.31 | 0.19 | 0.30 | 0.29 | 0.34 | 0.35 | 0.38 | 0.35 |
| MP2 | 0.07 | 0.18 | 0.24 | 0.22 | 0.21 | 0.23 | 0.28 | 0.25 | 0.23 | 0.29 | 0.31 | 0.28 | 0.26 | 0.28 |
| MP3 | 0.07 | 0.26 | 0.18 | 0.26 | 0.25 | 0.23 | 0.27 | 0.17 | 0.23 | 0.22 | 0.23 | 0.30 | 0.25 | 0.27 |
| MP4 | 0.07 | 0.12 | 0.13 | 0.17 | 0.28 | 0.26 | 0.21 | 0.24 | 0.29 | 0.31 | 0.33 | 0.33 | 0.29 | 0.30 |
| MP5 | 0.07 | 0.36 | 0.24 | 0.27 | 0.21 | 0.23 | 0.22 | 0.28 | 0.20 | 0.19 | 0.24 | 0.20 | 0.22 | 0.21 |
| MP6 | 0.15 | 0.22 | 0.24 | 0.18 | 0.29 | 0.27 | 0.22 | 0.21 | 0.34 | 0.36 | 0.34 | 0.38 | 0.30 | 0.32 |
| CS1 | 0.15 | 0.27 | 0.19 | 0.18 | 0.34 | 0.36 | 0.34 | 0.18 | 0.38 | 0.37 | 0.35 | 0.39 | 0.46 | 0.40 |
| CS2 | 0.06 | 0.22 | 0.24 | 0.26 | 0.36 | 0.32 | 0.28 | 0.20 | 0.39 | 0.35 | 0.40 | 0.40 | 0.37 | 0.35 |
| CS3 | 0.15 | 0.17 | 0.14 | 0.13 | 0.36 | 0.40 | 0.22 | 0.14 | 0.41 | 0.47 | 0.41 | 0.38 | 0.45 | 0.39 |
| CS4 | 0.15 | 0.18 | 0.20 | 0.14 | 0.34 | 0.37 | 0.23 | 0.18 | 0.39 | 0.38 | 0.36 | 0.37 | 0.40 | 0.37 |
| CS5 | 0.13 | 0.23 | 0.30 | 0.15 | 0.45 | 0.49 | 0.19 | 0.22 | 0.48 | 0.47 | 0.46 | 0.47 | 0.50 | 0.50 |
| CS6 | 0.15 | 0.23 | 0.14 | 0.23 | 0.57 | 0.53 | 0.23 | 0.14 | 0.56 | 0.55 | 0.57 | 0.54 | 0.58 | 0.55 |
| CS7 | 0.12 | 0.18 | 0.23 | 0.18 | 0.48 | 0.45 | 0.27 | 0.14 | 0.44 | 0.49 | 0.45 | 0.49 | 0.46 | 0.46 |
| SC1 | 0.25 | 0.18 | 0.20 | 0.22 | 0.62 | 0.64 | 0.28 | 0.11 | 0.61 | 0.68 | 0.67 | 0.62 | 0.64 | 0.63 |
| SC2 | 0.18 | 0.22 | 0.19 | 0.10 | 0.58 | 0.63 | 0.18 | 0.14 | 0.60 | 0.61 | 0.57 | 0.58 | 0.63 | 0.62 |
| SC3 | 0.11 | 0.17 | 0.14 | 0.20 | 0.66 | 0.69 | 0.22 | 0.13 | 0.56 | 0.55 | 0.60 | 0.66 | 0.68 | 0.64 |
| SC4 | 0.18 | 0.22 | 0.15 | 0.18 | 0.64 | 0.63 | 0.18 | 0.17 | 0.63 | 0.58 | 0.60 | 0.61 | 0.63 | 0.65 |
| SC5 | 0.17 | 0.20 | 0.14 | 0.13 | 0.53 | 0.61 | 0.21 | 0.10 | 0.57 | 0.53 | 0.53 | 0.53 | 0.64 | 0.54 |
| SC6 | 0.16 | 0.19 | 0.20 | 0.15 | 0.55 | 0.60 | 0.15 | 0.14 | 0.59 | 0.60 | 0.57 | 0.58 | 0.60 | 0.61 |
| SC7 | 0.17 | 0.17 | 0.18 | 0.17 | 0.61 | 0.67 | 0.22 | 0.13 | 0.65 | 0.64 | 0.61 | 0.61 | 0.66 | 0.62 |
| FS1 | 0.17 | 0.23 | 0.22 | 0.20 | 0.59 | 0.64 | 0.21 | 0.13 | 0.65 | 0.69 | 0.63 | 0.67 | 0.67 | 0.68 |
| FS2 | 0.11 | 0.13 | 0.19 | 0.17 | 0.70 | 0.70 | 0.22 | 0.13 | 0.62 | 0.58 | 0.66 | 0.73 | 0.66 | 0.68 |
| FS3 | 0.17 | 0.17 | 0.22 | 0.17 | 0.63 | 0.66 | 0.17 | 0.10 | 0.62 | 0.68 | 0.65 | 0.69 | 0.68 | 0.61 |
| FS4 | 0.19 | 0.19 | 0.16 | 0.15 | 0.67 | 0.73 | 0.19 | 0.12 | 0.75 | 0.67 | 0.67 | 0.71 | 0.73 | 0.69 |
| FS5 | 0.16 | 0.16 | 0.17 | 0.16 | 0.58 | 0.66 | 0.16 | 0.10 | 0.62 | 0.63 | 0.57 | 0.58 | 0.69 | 0.59 |
| FS6 | 0.11 | 0.17 | 0.22 | 0.17 | 0.66 | 0.69 | 0.17 | 0.13 | 0.59 | 0.60 | 0.62 | 0.66 | 0.71 | 0.73 |
| **WBA** | **0.67** | **0.69** | **0.66** | **0.75** | **-0.67** | **-0.48** | **0.58** | **0.59** | **0.46** | **0.48** | **0.57** | **-0.45** | **0.62** | **0.56** |

|  | MP1 | MP2 | MP3 | MP4 | MP5 | MP6 | CS1 | CS2 | CS3 | CS4 | CS5 | CS6 | CS7 |
| --- | --- | --- | --- | --- | --- | --- | --- | --- | --- | --- | --- | --- | --- |
| MP1 | 1.00 |  |  |  |  |  |  |  |  |  |  |  |  |
| MP2 | 0.35 | 1.00 |  |  |  |  |  |  |  |  |  |  |  |
| MP3 | 0.44 | 0.35 | 1.00 |  |  |  |  |  |  |  |  |  |  |
| MP4 | 0.38 | 0.34 | 0.29 | 1.00 |  |  |  |  |  |  |  |  |  |
| MP5 | 0.25 | 0.22 | 0.17 | 0.21 | 1.00 |  |  |  |  |  |  |  |  |
| MP6 | 0.40 | 0.36 | 0.44 | 0.30 | 0.18 | 1.00 |  |  |  |  |  |  |  |
| CS1 | 0.46 | 0.32 | 0.36 | 0.31 | 0.18 | 0.41 | 1.00 |  |  |  |  |  |  |
| CS2 | 0.37 | 0.37 | 0.36 | 0.32 | 0.18 | 0.49 | 0.42 | 1.00 |  |  |  |  |  |
| CS3 | 0.45 | 0.31 | 0.30 | 0.34 | 0.22 | 0.45 | 0.55 | 0.37 | 1.00 |  |  |  |  |
| CS4 | 0.36 | 0.38 | 0.37 | 0.36 | 0.18 | 0.47 | 0.53 | 0.47 | 0.52 | 1.00 |  |  |  |
| CS5 | 0.35 | 0.27 | 0.31 | 0.26 | 0.27 | 0.40 | 0.49 | 0.43 | 0.44 | 0.46 | 1.00 |  |  |
| CS6 | 0.36 | 0.32 | 0.31 | 0.35 | 0.18 | 0.46 | 0.52 | 0.42 | 0.60 | 0.53 | 0.62 | 1.00 |  |
| CS7 | 0.36 | 0.21 | 0.32 | 0.28 | 0.14 | 0.44 | 0.49 | 0.36 | 0.44 | 0.42 | 0.55 | 0.57 | 1.00 |
| SC1 | 0.33 | 0.30 | 0.25 | 0.32 | 0.22 | 0.42 | 0.42 | 0.37 | 0.49 | 0.44 | 0.54 | 0.66 | 0.56 |
| SC2 | 0.37 | 0.22 | 0.29 | 0.28 | 0.18 | 0.37 | 0.46 | 0.33 | 0.48 | 0.43 | 0.56 | 0.57 | 0.55 |
| SC3 | 0.35 | 0.28 | 0.23 | 0.30 | 0.20 | 0.31 | 0.39 | 0.31 | 0.38 | 0.33 | 0.43 | 0.50 | 0.43 |
| SC4 | 0.37 | 0.22 | 0.36 | 0.32 | 0.22 | 0.41 | 0.42 | 0.37 | 0.45 | 0.43 | 0.56 | 0.65 | 0.52 |
| SC5 | 0.38 | 0.20 | 0.26 | 0.26 | 0.23 | 0.30 | 0.53 | 0.28 | 0.44 | 0.39 | 0.48 | 0.53 | 0.56 |
| SC6 | 0.32 | 0.28 | 0.28 | 0.33 | 0.18 | 0.35 | 0.36 | 0.37 | 0.42 | 0.47 | 0.51 | 0.56 | 0.47 |
| SC7 | 0.31 | 0.24 | 0.27 | 0.27 | 0.24 | 0.32 | 0.44 | 0.32 | 0.43 | 0.41 | 0.57 | 0.55 | 0.52 |
| FS1 | 0.38 | 0.30 | 0.30 | 0.32 | 0.20 | 0.37 | 0.42 | 0.36 | 0.44 | 0.39 | 0.51 | 0.60 | 0.47 |
| FS2 | 0.31 | 0.28 | 0.27 | 0.34 | 0.21 | 0.35 | 0.36 | 0.35 | 0.39 | 0.41 | 0.50 | 0.55 | 0.46 |
| FS3 | 0.31 | 0.28 | 0.27 | 0.33 | 0.20 | 0.38 | 0.47 | 0.40 | 0.42 | 0.44 | 0.53 | 0.54 | 0.52 |
| FS4 | 0.35 | 0.27 | 0.26 | 0.33 | 0.23 | 0.35 | 0.44 | 0.35 | 0.51 | 0.45 | 0.56 | 0.64 | 0.51 |
| FS5 | 0.33 | 0.23 | 0.26 | 0.25 | 0.20 | 0.30 | 0.38 | 0.33 | 0.44 | 0.39 | 0.48 | 0.52 | 0.49 |
| FS6 | 0.35 | 0.27 | 0.27 | 0.30 | 0.20 | 0.31 | 0.36 | 0.34 | 0.38 | 0.40 | 0.50 | 0.54 | 0.46 |
| **WBA** | **0.73** | **0.68** | **0.65** | **0.64** | **0.73** | **0.72** | **0.56** | **0.49** | **0.41** | **0.49** | **0.51** | **0.42** | **0.53** |

|  | SC1 | SC2 | SC3 | SC4 | SC5 | SC6 | SC7 | FS1 | FS2 | FS3 | FS4 | FS5 | FS6 |
| --- | --- | --- | --- | --- | --- | --- | --- | --- | --- | --- | --- | --- | --- |
| SC1 | 1.00 |  |  |  |  |  |  |  |  |  |  |  |  |
| SC2 | 0.63 | 1.00 |  |  |  |  |  |  |  |  |  |  |  |
| SC3 | 0.59 | 0.55 | 1.00 |  |  |  |  |  |  |  |  |  |  |
| SC4 | 0.66 | 0.71 | 0.58 | 1.00 |  |  |  |  |  |  |  |  |  |
| SC5 | 0.58 | 0.63 | 0.53 | 0.63 | 1.00 |  |  |  |  |  |  |  |  |
| SC6 | 0.62 | 0.59 | 0.55 | 0.61 | 0.54 | 1.00 |  |  |  |  |  |  |  |
| SC7 | 0.63 | 0.68 | 0.59 | 0.65 | 0.65 | 0.61 | 1.00 |  |  |  |  |  |  |
| FS1 | 0.70 | 0.63 | 0.62 | 0.65 | 0.55 | 0.66 | 0.62 | 1.00 |  |  |  |  |  |
| FS2 | 0.66 | 0.59 | 0.73 | 0.62 | 0.54 | 0.61 | 0.62 | 0.62 | 1.00 |  |  |  |  |
| FS3 | 0.65 | 0.64 | 0.58 | 0.61 | 0.59 | 0.63 | 0.70 | 0.64 | 0.64 | 1.00 |  |  |  |
| FS4 | 0.70 | 0.69 | 0.65 | 0.69 | 0.63 | 0.68 | 0.72 | 0.75 | 0.69 | 0.71 | 1.00 |  |  |
| FS5 | 0.60 | 0.62 | 0.55 | 0.59 | 0.62 | 0.55 | 0.64 | 0.64 | 0.62 | 0.61 | 0.68 | 1.00 |  |
| FS6 | 0.62 | 0.61 | 0.66 | 0.64 | 0.53 | 0.63 | 0.61 | 0.67 | 0.70 | 0.60 | 0.68 | 0.61 |  |
| **WBA** | **0.71** | **0.68** | **0.64** | **-0.61** | **0.68** | **0.65** | **0.68** | **0.63** | **-0.58** | **0.55** | **0.67** | **0.60** | **-0.56** |

**Table A5. Factor Loadings and Cross-Loadings in six-factor WBA Model Based on the T1 and T2 Stacked File.**

| Item code | Emotional Health | Physical Health | Meaning and Purpose | Social Connectedness | Character Strengths | Financial Security |
| --- | --- | --- | --- | --- | --- | --- |
| EH1 | **0.659*** | 0.035 | 0.040 | 0.122* | 0.002 | 0.041* |
| EH2 | **0.719*** | 0.017 | 0.014 | 0.103* | 0.006 | 0.005 |
| EH3 | **0.512*** | 0.005 | 0.137* | 0.063* | 0.116* | 0.003 |
| EH4 | **0.789*** | -0.014 | 0.048* | 0.000 | 0.079* | 0.027* |
| EH5 | **-0.712*** | -0.04* | 0.023 | -0.054* | 0.052* | -0.023 |
| EH6 | **-0.524*** | -0.035 | 0.021 | 0.079* | 0.001 | -0.020 |
| EH7 | **0.488*** | 0.010 | 0.036 | -0.127* | 0.395* | 0.003 |
| PH1 | 0.241* | **0.424*** | 0.003 | -0.033 | 0.093* | 0.118* |
| PH2 | -0.049* | **0.797*** | -0.041 | 0.030 | -0.037* | 0.018 |
| PH3 | 0.018 | **0.691*** | -0.025 | -0.061* | 0.063* | 0.022 |
| PH4 | -0.014 | **0.848*** | 0.019 | 0.040* | -0.030* | -0.028* |
| PH5 | -0.081* | **-0.630*** | -0.005 | -0.007 | 0.091* | 0.049* |
| PH6 | -0.003 | **0.815*** | 0.041 | 0.057* | 0.045* | -0.008 |
| PH7 | 0.106* | **0.423*** | 0.069* | -0.028 | 0.139* | 0.112* |
| MP1 | 0.095* | 0.020 | **0.531*** | 0.018 | 0.234* | -0.034* |
| MP2 | 0.102* | 0.003 | **0.665*** | 0.043* | 0.083* | -0.064* |
| MP3 | 0.025 | 0.016 | **0.975*** | -0.015 | -0.040* | 0.007 |
| MP4 | -0.030* | 0.012 | **0.987*** | -0.016 | -0.029* | 0.035* |
| MP5 | 0.483* | 0.001 | **0.318*** | 0.073* | 0.039* | -0.027* |
| MP6 | 0.17* | -0.01 | **0.521*** | 0.095* | 0.093* | 0.049* |
| SC1 | 0.106* | -0.012 | 0.013 | **0.733*** | 0.047* | 0.033* |
| SC2 | 0.028 | -0.004 | 0.029 | **0.750*** | 0.041* | -0.003 |
| SC3 | -0.469* | -0.011 | -0.056 | **-0.274*** | 0.126* | 0.003 |
| SC4 | 0.031 | -0.002 | -0.056* | **0.971*** | -0.006 | -0.056* |
| SC5 | -0.012 | 0.038* | 0.07* | **0.731*** | -0.014 | 0.041* |
| SC6 | 0.015 | 0.047* | 0.233* | **0.447*** | 0.074* | 0.095* |
| SC7 | -0.016 | 0.045* | 0.153* | **0.375*** | 0.172* | 0.109* |
| CS1 | 0.148* | 0.026 | 0.043 | 0.040 | **0.633*** | -0.030* |
| CS2 | 0.044 | -0.011 | 0.131* | 0.021 | **0.491*** | 0.093* |
| CS3 | 0.016 | 0.010 | -0.082* | 0.078* | **0.704*** | -0.067* |
| CS4 | -0.003 | 0.032 | 0.002 | 0.015 | **0.697*** | 0.052* |
| CS5 | 0.029 | 0.012 | -0.023 | -0.020 | **0.814*** | 0.006 |
| CS6 | -0.088* | -0.004 | 0.064* | 0.023 | **0.705*** | -0.002 |
| FS1 | -0.021 | -0.003 | 0.212* | 0.097* | **0.508*** | 0.007 |
| FS2 | 0.106* | -0.009 | -0.011 | 0.043* | 0.047* | **0.730*** |
| FS3 | -0.223* | -0.075* | 0.038 | 0.003 | 0.078* | **-0.560*** |
| FS4 | -0.06* | -0.001 | -0.002 | -0.009 | 0.012 | **0.897*** |
| FS5 | 0.003 | -0.027* | 0.064* | 0.039* | -0.007 | **0.906*** |
| FS6 | -0.028 | 0.051* | 0.054* | 0.018 | 0.006 | **0.812*** |
| FS7 | -0.145* | 0.006 | 0.066* | 0.022 | 0.012 | **-0.693*** |

*Note:* * indicates the estimates significant at at least p<0.05. Item codes correspond to item wording presented in Table1 in the manuscript.

**Table A6. Goodness of Fit for Time Measurement Invariance of Six One-Factor Models Based on the T1 and T2 Stacked File.**

| Model | CFI | TLI | RMSEA |
| --- | --- | --- | --- |
| Emotional Health one-factor CFA+covariance between the error terms^a^ |  |  |  |
| *configural* | 0.933 | 0.891 | 0.106 |
| *metric* | 0.931 | 0.910 | 0.097 |
| *scalar* | 0.917 | 0.909 | 0.097 |
| Emotional Health 2 factor ESEM |  |  |  |
| *configural* | 0.969 | 0.919 | 0.092 |
| *metric* | 0.973 | 0.956 | 0.068 |
| *scalar* | 0.971 | 0.961 | 0.064 |
| Physical Health one-factor CFA - limited item set^b^ |  |  |  |
| *configural* | 0.968 | 0.947 | 0.069 |
| *metric* | 0.966 | 0.956 | 0.063 |
| *scalar* | 0.875 | 0.866 | 0.110 |
| Physical Health 2 factor ESEM |  |  |  |
| *configural* | 0.983 | 0.955 | 0.060 |
| *metric* | 0.983 | 0.972 | 0.047 |
| *scalar* | 0.958 | 0.943 | 0.068 |
| Meaning and Purpose one-factor CFA |  |  |  |
| *configural* | 0.932 | 0.899 | 0.109 |
| *metric* | 0.929 | 0.913 | 0.101 |
| *scalar* | 0.921 | 0.917 | 0.099 |
| Meaning and Purpose 2 factor ESEM |  |  |  |
| *configural* | 0.973 | 0.929 | 0.091 |
| *metric* | 0.974 | 0.957 | 0.071 |
| *scalar* | 0.970 | 0.959 | 0.069 |
| Character Strengths |  |  |  |
| *configural* | 0.962 | 0.942 | 0.069 |
| *metric* | 0.959 | 0.949 | 0.065 |
| *scalar* | 0.948 | 0.946 | 0.067 |
| Social Connectedness one-factor CFA +covariance between the error terms^c^ |  |  |  |
| *configural* | 0.970 | 0.952 | 0.076 |
| *metric* | 0.968 | 0.958 | 0.071 |
| *scalar* | 0.963 | 0.959 | 0.070 |
| Social Connectedness 2 factor ESEM |  |  |  |
| *configural* | 0.988 | 0.970 | 0.060 |
| *metric* | 0.986 | 0.978 | 0.052 |
| *scalar* | 0.983 | 0.977 | 0.053 |
| Financial Security one-factor CFA+covariance between the error terms^d^ |  |  |  |
| *configural* | 0.964 | 0.933 | 0.109 |
| *metric* | 0.961 | 0.944 | 0.100 |
| *scalar* | 0.955 | 0.948 | 0.096 |
| Financial Security 2 factor ESEM |  |  |  |
| *configural* | 0.995 | 0.980 | 0.060 |
| *metric* | 0.993 | 0.986 | 0.049 |
| *scalar* | 0.991 | 0.987 | 0.048 |
| WBA 6 factor ESEM+covariance of error terms^e^ |  |  |  |
| *configural* | 0.941 | 0.916 | 0.047 |
| *metric* | 0.940 | 0.929 | 0.043 |
| *scalar* | 0.938 | 0.918 | 0.046 |

*Note:* ^a^ covariance between error terms of two negatively oriented items in the emotional health domain: depression and anxiety; ^b^ without the item ‘I regularly do things to maintain and improve my health, in diet, exercise and healthcare’; ^c^ covariance between error terms of the only two items refereeing particularly to participant’s broader community: ‘I feel connected to the broader community around’ and ‘People in my broader community trust and respect one another’;
^d^ covariance between error terms of the only two negatively oriented items: ‘How often do you worry about food, housing, or health expenses?’ And ‘The amount of debt I have often overwhelms me’; ^e^ covariance between error terms of (1) two negatively oriented items in the financial security domain; (2) two negatively oriented items in the emotional health domain; and (3) the only two items referring particularly to a participant’s broader community in the social connectedness domain; detailed factor loading structure with cross loadings is presented in the Supplementary Material 2, Table A4.
